# Attentional disengagement effect based on relevant features

**DOI:** 10.3389/fpsyg.2022.960183

**Published:** 2022-11-09

**Authors:** Yuxiang Hao, Qi Zhang, Zile Wang, Mengxuan Sun

**Affiliations:** School of Educational Science, Minnan Normal University, Zhangzhou, China

**Keywords:** attentional capture, visual search, attentional disengagement, saccadic latency, eye movements

## Abstract

In visual search tasks, distractors similar to the target can attract our attention and affect the speed of attentional disengagement. The attentional disengagement refers to shifting attention away from stimuli that are not relevant to the task. Previous studies mainly focused on the attentional disengagement of one feature dimension. However, the mechanisms of different feature dimensions on attentional disengagement in single and conjunction visual search remain unclear. In the current study, we adopted the oculomotor disengagement paradigm and used saccade latency as an indicator to explore the effects of different feature dimensions of center stimuli on attentional disengagement. In both single and conjunction feature search tasks, participants began each search by fixating on a center stimulus that appeared simultaneously with search display but would not be the target. Participants were instructed to ensure the first saccade to the target location. In Experiments 1A (single feature search) and 1B (conjunction feature search), we found that the attentional disengagement was significantly delayed or accelerated when center stimuli shared color features with the target or salient distractor, but not in shape feature. Moreover, we found that the difference between the two feature dimensions might be caused by their different search difficulty (Experiment 1C). Therefore, in Experiment 2, we matched the difficulty of searching for color and shape tasks before exploring whether there were differences in the effects of different feature dimensions on attentional disengagement. However, the results in Experiment 2 were similar to those in Experiment 1A, indicating that the different effects of feature dimensions on attentional disengagement were caused by feature asymmetry. Therefore, in Experiment 3, we improved the salient discernibility of shape dimension and matched color search to it. The results showed that although the attentional disengagement was delayed in shape dimension, it was still smaller than that in color dimension. Our results supported that goal-oriented attention sets were the main cause of delayed attentional disengagement. By series of experiments, we found that the utilization of different feature dimensions was associated with task difficulty and the features asymmetry in both single and conjunction visual search.

## Introduction

In our daily life, we are often surrounded by a wealth of information, therefore attentional selection is important for us. Due to the limitation of cognitive resources, the attentional selection mechanism can help us to filter and exclude irrelevant or unimportant information. However, some irrelevant information can still capture our attention. In visual search tasks, attention could be captured by the task-irrelevant stimulus that was similar to the target even though the target would not be present at that location or time (Folk et al., 1992, 2002, 2008; Folk and Remington, 1998). This type of capture has been named contingent capture, its processing mechanism was a top-down process triggered by our goals and intentions (Folk et al., 1992, 1994; Folk and Remington, 1998). The salient or unique stimuli could also capture attention because bottom-up salience-driven process tended to direct attention to salient stimuli in the environment (Theeuwes et al., 1999, 2003, 2004; Franconeri and Simons, 2003; Theeuwes, 2010; Kerzel and Schönhammer, 2013). Theeuwes (2010) proposed that the initial visual selection was driven by the stimulus, and volitional control based on expectancy and goal set after feedback processing will bias visual selection in a top-down manner (i.e., rapid disengagement hypothesis). Currently, many studies provided experimental support for the rapid disengagement hypothesis (Brockmole and Boot, 2009; Geng and Di Quattro, 2010; Blakely et al., 2012; Wright et al., 2015a,b; Schoeberl et al., 2019; Wang et al., 2019). Disengagement has been discussed as a means to distinguish between bottom-up and top-down theories of attention capture (Blakely et al., 2012). However, a neuroimaging study showed that although there was clearly a strong correlation association between goal-directed orienting and superior parietal lobule (SPL), stimulus-driven orienting and temporoparietal junction (TPJ), the two systems were not entirely independent (Shomstein et al., 2010). Top-down signals facilitated attentional guidance towards behaviorally relevant locations and features (Baluch and Itti, 2011). Lamy and Kristjánsson (2013) shared the same view and suggested that goal-directed signals depend on the degree of match between an object and the set of target attributes specified by task demands. However, Anderson’s (2014) study suggested that goal-directed attentional selection may be imprecise, and participants’ ability to select targets among feature-similar non-targets was impaired when stimuli and target templates were compared continuously in time. In other words, distractors with target-defining attribute may capture attention and slow down target identification (Ghorashi et al., 2003).

Although there are a lot of research on attentional capture, the mechanism of attentional capture is still controversial. The attentional capture paradigm of previous behavioral studies mainly included the spatial cueing paradigm, the additional singleton paradigm, and their variants (Folk et al., 1992; Anderson and Folk, 2010; Belopolsky et al., 2010; Gaspelin and Luck, 2018; Wang et al., 2019). These paradigms used the reaction time as an indicator, so it was difficult to distinguish whether the loss of reaction time was due to the distractor attracting attention to a location or to maintaining attention at that location (Blakely et al., 2012). The oculomotor disengagement paradigm used saccadic latency as an indicator to intuitively explore the duration of attention held on the center distractor (Brockmole and Boot, 2009; Boot and Brockmole, 2010; Wright et al., 2015a,b). In addition, some studies have explored attentional disengagement in social contexts through the eye movement disengagement paradigm and its variants. Dalmaso et al. (2019) investigated whether shapes associated with the self could hold attention. It turns out that when the shape was related to the self instead of a stranger, the speed of the participants away from the center shape would slow down. The response times of saccade to left and right targets were influenced by the type of central face (angry or happy), with angry faces disengaging slower than neutral or happy faces (Belopolsky et al., 2011). Some studies using the disengagement paradigm showed that the direction of gaze or eye contact of facial stimuli could affect saccade response time (Ueda et al., 2014; Dalmaso et al., 2017). For different populations, the attention of patients with high anxiety was easily guided by threatening social stimuli (Azarian et al., 2016a,b). The saccadic latency was defined as the time between the presentation of the search display and the first saccade away from the center stimuli (Boot and Brockmole, 2010). Participants began each search by fixating on a center stimulus in this paradigm. The target was always on the imaginary circle around the center stimulus. The center stimulus appeared simultaneously with the search display but would not be the target and was task-irrelevant.

Brockmole and Boot (2009) used the oculomotor disengagement paradigm and found that the saccadic latency to peripheral stimuli was delayed when the center stimulus was singleton and novel. The result showed that the features of irrelevant stimuli influence the speed of attentional disengagement. Boot and Brockmole (2010) further found that attentional disengagement was delayed when the center stimulus was the same color as the search target, compared to other colors, regardless of whether the center stimulus and the search display were presented simultaneously or with intervals (Boot and Brockmole, 2010). Blakely et al. (2012) replicated the experiment of Boot and Brockmole (2010) and further explored the time course of the attentional disengagement effect. They found that the delayed disengagement effect increased as the interstimulus interval between the presentation of peripheral and center stimuli decreased and it was greatest when the center stimulus was presented simultaneously with the peripheral stimuli. Blakely et al. (2012) also observed delayed attentional disengagement in single-feature search of shape dimension. In addition, a previous study showed that attentional delay disengagement was functional significance, automatically encouraging deeper processing of stimuli similar to the target (Wright et al., 2015a). These studies can help us better understand the factors that influence attentional disengagement.

For attentional disengagement, previous studies mainly focused on one feature dimension and tested it in single-feature search (Brockmole and Boot, 2009; Boot and Brockmole, 2010; Blakely et al., 2012). However, the mechanisms of different feature dimensions on attentional disengagement are still unclear. Extensive research showed that there was an asymmetry between different feature dimensions (Zohary and Hochstein, 1989; Poisson and Wilkinson, 1992; Moutoussis and Zeki, 1997; Hannus et al., 2006). A previous study showed that participants were more quickly aware of color differences when color and shape were presented simultaneously in a search display, and the selection of attention in visual search depends on the relative discriminability of the stimuli feature dimensions (Theeuwes, 1992). The brain combined features based on perceived simultaneously rather than occurring simultaneously, and experiments showed that color feature was preferentially perceived when presented simultaneously with motion (Moutoussis and Zeki, 1997). In the feature preview task, the color preview had an advantage over the orientation preview. Participants were more likely to group stimuli by color features in conjunction feature search (Olds and Fockler, 2004; Sobel et al., 2009). Although the feature asymmetry has been extensively studied, its effect on attentional disengagement was unclear.

In the current study, the target was defined by both color and shape in single and conjunction feature search tasks. However, the degree of feature utilization was different in the two search tasks. Participants can find the target by using one feature in single-feature search, while must utilize two features to identify the target in conjunction feature search. Therefore, we adopted the oculomotor disengagement paradigm and used saccade latency as an indicator to explore the effects of different feature dimensions of center stimuli on attentional disengagement in both single and conjunction feature search tasks. In Experiments 1A and 1B, we found that the color feature and shape feature of the center stimulus have different effects on attentional disengagement in single and conjunction feature searches. Therefore, we further investigated whether there was a difference in difficulty between color and shape feature dimensions in Experiment 1C. In Experiments 2 to 3, we matched the task difficulty of searching for color and shape before exploring the attentional disengagement effects of different feature dimensions.

## Experiment 1A

### Method

#### Participants

Thirty-six healthy young adults (34 females; age (mean ± SD): 20.92 ± 1.57 years; age range: 18-25 years) participated in Experiment 1A. All participants had normal or corrected-to-normal color vision. All participants signed an informed consent form and were naive to the purpose of the experiment. Each participant was paid for the experiment. The research was approved by the ethics committee.

#### Materials and design

**FIGURE 1:**
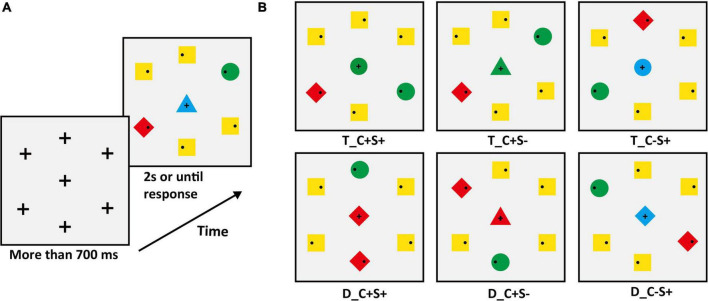
**(A)** Schematic procedure of Experiment 1A (e.g., target: green circle). The second search display was the baseline condition. **(B)** The condition of the center stimuli. T_ = the center stimulus shares features with the target, D_ = center stimulus shares features with salient distractor; C = color, S = shape; the “ + ” stands for same, “–” stands for different.

The search display consisted of seven stimuli on a gray (RGB: 127, 127, 127) background with *a* luminance level of 75 cd/m^2^. One stimulus was located at the center of the display. Six stimuli were equidistantly spaced around an imaginary circle with a diameter of 7.8°. Inside peripheral stimuli, each contained a small black dot (diameter: 0.26°; RGB: [0, 0, 0]) that was randomly distributed on the left or right. The peripheral stimuli consisted of a target (circle) and four non-salient distractors (squares) and a salient distractor (diamond). The experiments matched the area of different shapes. The target color was red (RGB: 255, 0, 0) or green (RGB: 0, 255, 0) and varied between subjects, the color of the non-salient distractor was yellow (RGB: 255, 255, 0), and the color of the salient distractor was opposite to the target color. For example, if the target color was green then the salient distractor color was red, and vice versa. The center stimulus varied between seven conditions (examples are shown in Figure 1), which were (1) identical to the target (T_C + S +); (2) identical to the color of the target and different in shape from the target, and all distractors (T_C + S–, shape: triangle); (3) identical to the shape of the target and different in color from target and all distractors (T_C–S, color: blue [RGB: 0, 0, 255]); (4) identical to the salient distractor (D_C + S +); (5) identical to the color of salient distractor and different in shape from target and all distractors (D_C + S–, shape: triangle); (6) identical to the shape of salient distractor and different in color from target and all distractors (D_C–S+, color: blue). For the baseline condition, the center stimulus was completely different from the target and distractor (Figure 1A). The letter “T” means that the center stimulus shares features with the target and “D” means that the center stimulus shares features with the distractor, “C” means color, and “S” means shape. The “ + ” stands for same and “–” stands for different. The center stimulus was presented simultaneously with the peripheral stimuli and the target was always within the peripheral circulation. The search display was presented for 2 s or until response.

#### Apparatus

All stimuli were displayed on a 37.5 × 30 cm monitor with a spatial resolution of 1,280 × 1,024 pixels and refresh a rate of 75 Hz. The stimuli were presented using Psychtoolbox3.0 (Brainard, 1997; Pelli, 1997) in MATLAB programming environment (MathWorks, Natick, MA, USA). Eye movements were recorded using an EyeLink1000 (SR Research, Ontario, Canada) eye tracker with a sampling rate of 1,000 Hz. We used nine-point calibration and validation procedure. Drift correction was performed before each block. An eye movement was classified as a saccade if its motion exceeded 0.1° and its acceleration reached 8,000 deg/s^2^ or its velocity reached 30 deg/s. The participants were positioned 63 cm to view the screen in a dimly lit room. We used a chin rest to fix the participants’ head position.

#### Procedure

The sequence of displays in a typical trial of search tasks was shown in Figure 1A (the target was a green circle). Each trial began with the display that consisted of a fixation cross (Length: 0.53°; Width: 0.13°; RGB: [0, 0, 0]) in the center and six crosses (Length: 0.53°; Width: 0.13°; RGB: [0, 0, 0]) equidistantly spaced around an imaginary circle. The position of the seven crosses corresponded to the position of the search display stimulus. To begin each trial, participants should fixate on the center cross in this display. The search display would not appear until the participants fixated on the center cross for more than 700 ms. Participants were instructed to ensure the first saccade to the target location as quickly as possible and then respond to whether the small black dot within the target was on the left or right side by pressing the left and right arrow keys while fixating on the target. The purpose of key response was to ensure that the participants completed the experiment carefully, and the trials with no response or incorrect responses and participants with less than 50% accuracy were excluded. The target was a red circle or green circle and varied between subjects. Participants were told that the center stimulus was irrelevant to the task. Target location varied randomly from trial to trial. The center stimulus was selected from the seven center stimuli conditions with equal proportions. All participants completed one practice block of 42 trials. The main experiment consisted of 12 blocks (42 trials in each block) for a total of 504 trials. Participants were given the option to rest at the end of each block.

### Results and discussion

#### Data analysis

Trials were excluded from the analysis if (a) the participant blinked or made a saccade at the time when the search display appeared, or the saccadic latency was less than 80 ms (mean ± SD: 7.01 ± 5.47%); (b) the location of the first saccade did not within 2° of target location (mean ± SD:14.26 ± 9.20%). Behavioral and eye movement data used the same exclusion criteria. Therefore, two participants were excluded from the analysis because the accuracy of the keypress response was less than 50%. The final sample size was 34. The accuracy of the keypress response was 98.32 ± 3.00%, and the mean RT of correct trials was 0.68 ± 0.11 s. Only correct trials were included in the analysis of eye movement data. We used the same trial exclusion criteria and data analysis methods in all experiments. The saccadic reaction time (SRT) (ms), reaction time (s), accuracy, and saccadic accuracy for all conditions of participants are shown in Table 1.

**TABLE 1:** Mean saccadic reaction time (ms) and reaction time (s) and accuracy and saccadic accuracy for all condition of Exp.1A (standard deviations are within parentheses).

	T_C + S +	T_C + S–	T_C–S +	Baseline	D_C + S +	D_C + S–	D_C–S +
SRT (ms)	267 (55)	261 (47)	243 (42)	241 (40)	236 (40)	236 (37)	242 (40)
RT (s)	0.69 (0.11)	0.69 (0.11)	0.68 (0.13)	0.68 (0.12)	0.68 (0.12)	0.68 (0.12)	0.67 (0.12)
Accuracy	0.98 (0.04)	0.99 (0.04)	0.99 (0.02)	0.98 (0.04)	0.98 (0.03)	0.98 (0.03)	0.98 (0.03)
Saccadic accuracy	0.77 (0.14)	0.78 (0.13)	0.79 (0.11)	0.80 (0.13)	0.79 (0.14)	0.79 (0.13)	0.78 (0.12)

#### Saccadic disengagement

**FIGURE 2:**
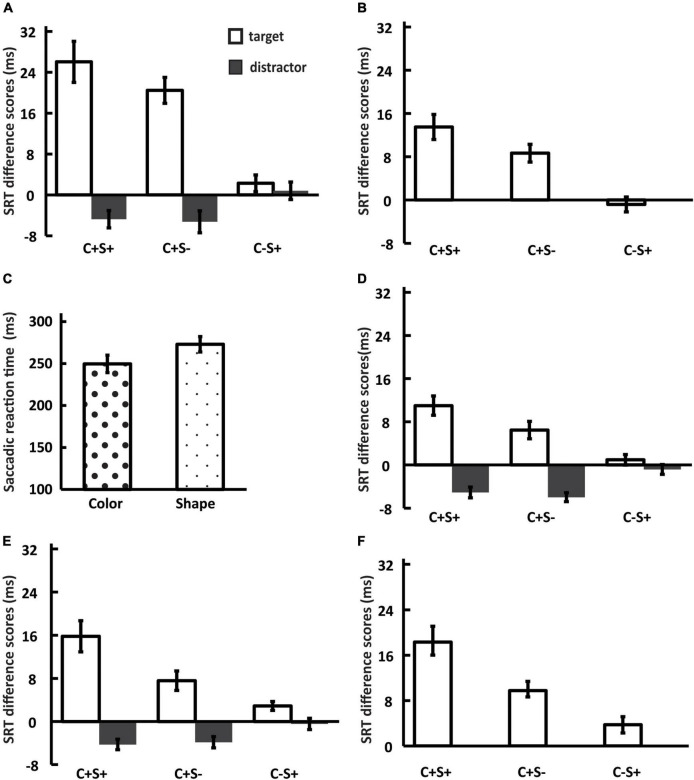
Results of SRT difference scores for Experiment 1A **(A)**, Experiment 1B **(B)**, Experiment 2 **(D)**, Experiment 3A **(E)**, and Experiment 3B **(F)**. **(C)** Results of SRT for color search task and shape search task (Experiment 1C). Error bars represent standard errors of the mean across participants. C + S + = consistent with target or distractor; C + S– = consistent with the color of target or distractor; C–S + = consistent with the shape of target or distractor.

We used the SRT as an indicator to analyze the saccadic disengagement. We subtracted the SRTs of the baseline condition from the SRTs of every other condition. The SRT difference scores were shown in Figure 2A. The 2 (center stimulus type: shared features with the target or salient distractor) × 3 (shared features: both color and shape, color, shape) repeated measures ANOVA (Bonferroni-corrected comparisons) was used to analyze the SRT differences scores. The results revealed significant main effect of center stimulus type (*F*(1, 33) = 64.08, *p* < 0.001, η*p*^2^ = 0.66), shared features (*F*(1.41, 46.48) = 13.67, *p* < 0.001, η*p*^2^ = 0.29), and their interaction (*F*(1.68, 55.29) = 29.30, *p* < 0.001, η*p*^2^ = 0.47). Further simple-effects analysis (Bonferroni-corrected comparisons) revealed that the SRT difference scores of the T_C + S + and the T_C + S– condition were significantly longer than that of the T_C-S + condition (T_C + S + : *t*(33) = 6.38, *p* < 0.001, Cohen’s *d* = 1.09, T_C + S–: *t*(33) = 7.88, *p* < 0.001, Cohen’s *d* = 1.35). No significant difference was found between the T_C + S + and the T_C + S– condition (*t*(33) = 2.15, *p* = 0.12, Cohen’s *d* = 0.37). Significant differences were found between the T_C + S + and the D_C + S + condition (*t*(33) = 8.34, *p* < 0.001, Cohen’s *d* = 1.43), between the T_C + S– and the D_C + S– condition (*t*(33) = 6.68, *p* < 0.001, Cohen’s *d* = 1.15). No significant difference was found between the T_C-S + and the D_C-S + condition (*t*(33) = 0.61, *p* = 0.55, Cohen’s *d* = 0.10). No significant differences were found between the D_C + S + and the D_C + S– condition (*t*(33) = 0.22, *p* = 1.00, Cohen’s *d* = 0.04), between the D_C + S + and the D_C-S + condition (*t*(33) = −2.32, *p* = 0.08, Cohen’s *d* = −0.40), and between the D_C + S– and the D_C-S + condition (*t*(33) = −2.27, *p* = 0.09, Cohen’s *d* = −0.39).

We further examined whether the attentional disengagement was delayed or accelerated by one-sample *t*-tests comparing the SRT difference scores to zero. The results revealed that significant delay attentional disengagement in the T_C + S + (*t*(33) = 6.49, *p* < 0.001, Cohen’s *d* = 1.11) and the T_C + S– condition (*t*(33) = 8.10, *p* < 0.001, Cohen’s *d* = 1.39). The attentional disengagement was significantly accelerated in the D_C + S + (*t*(33) = −2.81, *p* < 0.01, Cohen’s *d* = −0.48) and the D_C + S– condition (*t*(33) = −2.44, *p* < 0.05, Cohen’s *d* = −0.42). No significant difference was found in the T_C-S + (*t*(33) = 1.41, *p* = 0.17, Cohen’s *d* = 0.24) and the D_C-S + condition (*t*(33) = 0.48, *p* = 0.63, Cohen’s *d* = 0.08).

In the single-feature search task (Experiment 1A), we found that there was a significant delay in attentional disengagement when the center stimulus was the same color as the target, regardless of shape. And when the center stimulus shared color features with the salient distractor, attentional disengagement was accelerated. However, no significant effect was found on attentional disengagement for shape features. In the single-feature search (Experiment 1A), we found that the utilization of different feature dimensions was different, such as color and shape. Participants can find the target by using one feature in single-feature search, while in conjunction feature, search participants must utilize two features to identify the target. The effects of two feature dimensions on attentional disengagement in conjunction feature search were still unclear. Therefore, we further explored this question in Experiment 1B.

## Experiment 1B

### Method

#### Participants

Thirty-six healthy young adults (33 females; age (mean ± SD): 20.9 ± 1.57 years; age range: 18-25 years) participated in Experiment 1B. All participants had normal or corrected-to-normal color vision. All participants signed an informed consent form and were naive to the purpose of the experiment. Each participant was paid for the experiment. The research was approved by the ethics committee. The participants who took part in Experiment 1A except one who took part in Experiment 1B. An additional participant was recruited in Experiment 1B.

#### Materials and design

**FIGURE 3:**
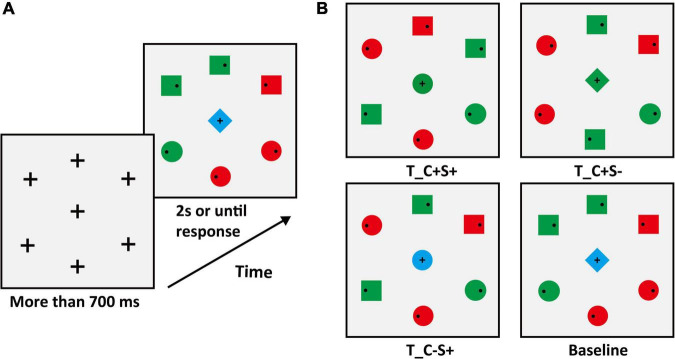
**(A)** Schematic procedure of Experiment 1B (e.g., target: green circle). **(B)** The condition of the center stimuli. T_ = target, D_ = distractor; C = color, S = shape; the “ + ” stands for same, “–” stands for different.

In Experiment 1B, we adopted the conjunction feature search task. The target color was red (RGB: 255, 0, 0) or green (RGB: 0, 255, 0) varying between subjects. The periphery of the search display consisted of three circles (one of them is the target) and three squares. Two of the three squares were the same color as the target. Another square and the two circles except the target were the opposite color to the target. Therefore, the colors of half periphery stimuli were red and the others were green (examples are shown in Figure 3). No salient distractors were set in Experiment 1B, so only the center stimulus types of sharing features with the target were considered. There were four conditions of the center stimulus (examples are shown in Figure 3B), which were (1) identical to the target (T_C + S +); (2) identical to the color of the target and different in shape from target and distractors (T_C + S–, shape: diamond); (3) identical to the shape of the target and different in color from target and distractors (T_C–S+, color: blue [RGB: 0, 0, 255]). For the baseline condition, the center stimulus was completely different from target and distractors. The center stimulus was presented simultaneously with the peripheral stimuli and the target was always within the peripheral circulation.

#### Apparatus and procedure

The apparatus parameters were the same as those in Experiment 1A. The sequence of displays in a typical trial of search tasks was shown in Figure 3A. Participants were instructed to ensure the first saccade to the target location as quickly as possible and then respond to whether the small black dot (Diameter: 0.26°; RGB: [0, 0, 0]) within the target was on the left or right by pressing the left and right arrow keys while fixating on the target. The target was a red circle or green circle and varied between subjects. Participants were told that the center stimulus was irrelevant to the task. Target location varied randomly from trial to trial. The center stimulus was selected from the four center stimuli conditions with equal proportions. All participants completed one practice block of 42 trials. The main experiment consisted of 12 blocks (42 trials in each block) for a total of 504 trials. Participants were given the option to rest at the end of each block.

### Results and discussion

#### Data analysis

About 6.02 ± 6.37% trials were excluded from the analysis because the participant blinked or made a saccade at the time when the search display appeared, or the saccadic latency was less than 80 ms. About 43.13 ± 13.67% trials were excluded from the analysis because the location of the first saccade did not within 2° of the target location. Two participants were excluded from the experiment, one because the accuracy of key press response was lower than 50% and the other because one block of behavioral data was not recorded. The final sample size was 34. The accuracy of the keypress response was 98.66 ± 1.89%, and the mean RT of correct trials was 0.65 ± 0.08 s. The SRT (ms), reaction time (s), accuracy, and saccadic accuracy for all conditions of participants are shown in Table 2.

**TABLE 2:** Mean saccadic reaction time (ms) and reaction time (s) and accuracy and saccadic accuracy for all condition of Exp. 1B (standard deviations are within parentheses).

	T_C + S +	T_C + S–	T_C–S +	Baseline
SRT (ms)	253 (35)	248 (35)	238 (34)	239 (36)
RT (s)	0.66 (0.08)	0.65 (0.08)	0.65 (0.08)	0.64 (0.08)
Accuracy	0.99 (0.01)	0.99 (0.02)	0.99 (0.03)	0.99 (0.02)
Saccadic accuracy	0.94 (0.06)	0.94 (0.07)	0.94 (0.06)	0.94 (0.07)

#### Saccadic disengagement

We subtracted the SRT of the baseline condition from the SRT of each condition. The SRT difference scores were shown in Figure 2B. One-way ANOVA of the SRT differences scores revealed significant main effect of the center stimulus type (*F*(2, 66) = 40.82, *p* < 0.001, η*p*^2^ = 0.55). *Post hoc* comparisons with Bonferroni correction revealed that the SRT differences scores of the T_C + S + condition were significantly longer than the T_C + S– and the T_C–S + condition (T_C + S–: *t*(33) = 2.90, *p* < 0.05, Cohen’s *d* = 0.50, T_C–S + : *t*(33) = 8.19, *p* < 0.001, Cohen’s *d* = 1.41). Significant difference was found between the T_C + S– and the T_C–S + condition (*t*(33) = 6.77, *p* < 0.001, Cohen’s *d* = 1.16). We further examined whether the attentional disengagement was delayed or accelerated by one-sample *t*-tests comparing the SRT difference scores to zero. The results revealed significant delay in attentional disengagement in the T_C + S + (*t*(33) = 5.92, *p* < 0.001, Cohen’s *d* = 1.02) and the T_C + S– condition (*t*(33) = 5.37, *p* < 0.001, Cohen’s *d* = 0.92). No significant difference was found in the T_C–S + condition (*t*(33) = −0.60 *p* = 0.55, Cohen’s *d* = −0.10).

The result of the conjunction feature search task (Experiment 1B) showed that there was a significant delay in attentional disengagement when the center stimulus was the same color as the target, regardless of shape. The delayed disengagement was the longest when the center stimulus was identical to the target. And when the center stimulus shared color features with the salient distractor, attentional disengagement was accelerated. In both single and conjunction feature search tasks (Experiment 1A and 1B), we found that attentional disengagement was significantly delayed or accelerated when center stimuli shared color features with the target or salient distractor. However, no significant effect was found on attentional disengagement for shape features. It was still unclear whether the difference between the two feature dimensions was caused by their different search difficulty. Therefore, we tested whether there was a significant difference in the search difficulty between color and shape feature dimensions in Experiment 1C.

## Experiment 1C

### Method

#### Participants

Another 34 healthy adults (29 females; age (mean ± SD): 20.50 ± 1.75 years; age range: 18-25 years) participated in Experiment 1C. All participants had normal or corrected-to-normal color vision. All participants signed an informed consent form and were naive to the purpose of the experiment. Each participant was paid for the experiment.

#### Materials and design

**FIGURE 4:**
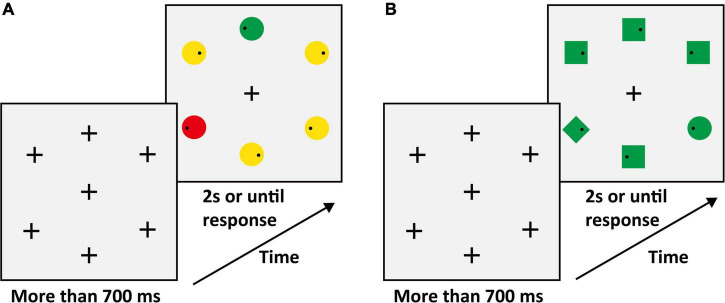
**(A)** Schematic procedure of the color search task (e.g., target: green circle). **(B)** Schematic procedure of the shape search task (e.g., target: green circle).

In Experiment 1C, we adopted the additional singleton paradigm. The participants were required to complete two tasks: a color search task and a shape search task. No center stimulus was presented in search tasks. In the color search task, the peripheral stimuli consisted of six circles, and the colors were the same as those in Experiment 1A. The target color was red or green and varied between subjects. In the shape search task, the shapes of peripheral stimuli were the same as those in Experiment 1A and the color was consistent with the target color of the color search task (examples are shown in Figure 4B, target: green circle).

#### Apparatus and procedure

The apparatus was the same as those in Experiment 1A. The sequence of displays in a typical trial of the matching phase was shown in Figure 4. Participants were instructed to ensure the first saccade to the target location as quickly as possible and respond to whether the small black dot (Diameter: 0.26°; RGB: [0, 0, 0]) within the target was on the left or right by pressing the left and right arrow keys. In the color search task, participants searched for a specific color. In the shape search task, participants searched for a specific shape. To balance the order effect, each participant took the order of ABBA or BAAB for different tasks. All participants completed one practice block of 10 trials in color search and shape search tasks, respectively. The main experiment consisted of four blocks (36 trials in each block) for a total of 144 trials. Participants were given the option to rest at the end of each block.

### Results and discussion

Trials were excluded from the analysis if (a) the participant blinked or made a saccade at the time when the search display appeared, or the saccadic latency was less than 80 ms (mean ± SD: color:8.37 ± 9.54%, shape:8.58 ± 7.22%) and (b) the location of the first saccade did not within 2° of target location (mean ± SD: color:27.33 ± 17.60%, shape:38.89 ± 18.63%). The accuracy of the keypress response for the color search task and shape search task were 99.56 ± 0.91% and 99.45 ± 1.34%, respectively. The mean RT of correct trials for color and shape search tasks were 0.69 ± 0.16 s and 0.74 ± 0.14 s, respectively.

A paired-samples *t*-test of the SRT for the color and shape search tasks revealed significant difference between searching for specific color (mean ± SD: 249.65 ± 59.59 ms) and shape (mean ± SD: 272.98 ± 52.53 ms), *t*(33) = –3.96, *p* < 0.001, Cohen’s *d* = –0.68 (see Figure 2C). And in color search, the proportion of trials in which the participants first correctly saccade to the target location was significantly higher than that in shape search (*t*(21) = 4.18, *p* < 0.001, Cohen’s *d* = 0.72). The results showed that there was a significant difference in the search difficulty between color and shape feature dimensions, and the saccade latency of searching for color was shorter than that of searching for shape. It was unclear whether the difference in the effects of two feature dimensions on attentional disengagement (the results of Experiment1A and 1B) was due to their search difficulty or due to the asymmetry of the features. Therefore, in Experiment 2, we matched the search difficulty of color and shape feature dimensions before exploring whether different feature dimensions had different effects on attentional disengagement. Moreover, a previous study has found feature asymmetry between the color and the shape feature dimensions (Theeuwes, 1992). If the two features still have different effects on attentional disengagement, we can exclude the effect of different search difficulties between the two features.

## Experiment 2

### Method

#### Participants

Another 34 healthy adults (26 females; age (mean ± SD): 20.37 ± 2.09 years; age range: 18-25 years) participated in Experiment 2. All participants had normal or corrected-to-normal color vision. Each participant signed an informed consent form.

#### Materials and design

To ensure that the search difficulty of color and shape feature dimensions was identical in the search task phase, we carried out a matching phase before the search task phase. The search display of the matching phase was similar to the search display of Experiment 1C (see Figure 4), except the color. Colors included red (RGB:175 + x, 175, 175), green (RGB:175, 175 + x, 175), yellow (RGB:175 + x, 175 + x, 175), and blue (RGB:175, 175, 175 + x). The color has six levels in the matching phase that correspond to x values of 5, 10, 15, 20, 25, and 30. With the increase of the value of “x”, discriminability between different colors increases. In the color search task of the matching phase, the color of each stimulus was randomly selected from the six levels with equal proportions. And participants were instructed to search for a circle that was redder or greener than the others. In the shape search task of the matching phase, participants were instructed to search for a circle. All stimuli were the same color and varied randomly among the six levels of the target color that were presented in the color search task. Each participant was required to complete the matching phase before the search task phase. To balance the order effect, each participant took the order of ABBA or BAAB for different tasks. We matched the level values of color according to the difficulty of the shape task. We adopted the Sigmoid curve fitting for the difficulty matching, and the formula was as follows:


$$f\,\left(x\right)=\mathrm{base}+\frac{\max}{1+\exp\,\left(\frac{\mathrm{xhalf}-x}{\mathrm{rate}}\right)}$$

The average SRT of shape search tasks was calculated for each participant (mean ± SD: 252.32 ± 27.09 ms). We calculated the SRTs of color search task under different color levels and gained the curve according to Sigmoid curve fitting. In this formula, the color level was served as x, and SRTs were served as y. After Sigmoid curve fitting, we used the curve and served average SRT of the shape search task which was y to calculate the color level for each participant (mean ± SD: 12.66 ± 5.93). This color level was used for stimuli in the search task phase.

#### Apparatus and procedure

The apparatus was the same as those in Experiment 1. The sequence of displays in a typical trial of the matching phase was the same as that in Experiment 1C. In the matching phase, participants completed one practice block of 72 trials in color search and shape search tasks, respectively. The color search task consisted of 10 blocks (36 trials in each block) for a total of 360 trials. The shape search task consisted of four blocks (36 trials in each block) for a total of 144 trials. In the search task phase, participants completed one practice block of 42 trials. The main experiment consisted of 12 blocks (42 trials in each block) for a total of 504 trials. Participants were given the option to rest at the end of each block.

### Results and discussion

#### Data analysis

About 4.36 ± 5.02% trials were excluded from the analysis because the participant blinked or made a saccade at the time when the search display appeared, or the saccadic latency was less than 80 ms. About 14.20 ± 9.05% trials were excluded from the analysis because the location of the first saccade did not within 2° of the target location. The accuracy of the keypress response was 95.65 ± 16.48%, and the mean RT of correct trials was 0.62 ± 0.07 s. The SRT (ms), reaction time (s), accuracy, and saccadic accuracy for all conditions of participants are shown in Table 3.

**TABLE 3:** Mean saccadic reaction time (ms) and reaction time (s) and accuracy and saccadic accuracy for all condition of Exp. 2 (standard deviations are within parentheses).

	T_C + S +	T_C + S–	T_C–S +	Baseline	D_C + S +	D_C + S–	D_C–S +
SRT (ms)	240 (21)	236 (20)	230 (17)	229 (17)	224 (18)	223 (16)	229 (17)
RT (s)	0.64 (0.08)	0.63 (0.07)	0.62 (0.07)	0.61 (0.07)	0.61 (0.08)	0.68 (0.07)	0.61 (0.08)
Accuracy	0.98 (0.01)	0.98 (0.02)	0.98 (0.02)	0.98 (0.02)	0.98 (0.02)	0.98 (0.02)	0.98 (0.03)
Saccadic accuracy	0.78 (0.13)	0.80 (0.14)	0.82 (0.13)	0.83 (0.11)	0.83 (0.10)	0.81 (0.13)	0.82 (0.11)

#### Saccadic disengagement

We subtracted the SRTs of the baseline condition from the SRT of every other condition. The SRT difference scores were shown in Figure 2D. The 2 (center stimulus type: shared features with the target or salient distractor) × 3 (shared features: both color and shape, color, shape) repeated measures ANOVA (Bonferroni-corrected comparisons) was used to analyze the SRT difference scores. The results revealed significant main effect of the center stimulus type (*F* (1, 33) = 91.13, *p* < 0.001, η*p*^2^ = 0.73), shared features (*F*(2, 66) = 6.64, *p* < 0.01, η*p*^2^ = 0.17), and their interaction (*F*(2, 66) = 26.60, *p* < 0.001, η*p*^2^ = 0.45). Further simple-effects analysis (Bonferroni-corrected comparisons) revealed that the SRT differences scores of the T_C + S + condition were significantly longer than that of the T_C + S– and the T_C–S + condition (T_C + S–: *t*(33) = 3.85, *p* < 0.01, Cohen’s *d* = 0.66, T_C–S + : *t*(33) = 5.38, *p* < 0.001, Cohen’s *d* = 0.92). The SRT difference scores of the T_C + S– condition were significantly longer than that of the T_C–S + condition (*t*(33) = 2.97, *p* < 0.05, Cohen’s *d* = 0.51). Significant differences were found between the T_C + S + and the D_C + S + condition (*t*(33) = 8.30, *p* < 0.001, Cohen’s *d* = 1.42), between the T_C + S– and the D_C + S– condition (*t*(33) = 7.43, *p* < 0.001, Cohen’s *d* = 1.28). No difference was found between the T_C–S + and the D_C–S + condition (*t*(33) = 1.83, *p* = 0.08, Cohen’s *d* = 0.31). When the center stimulus shared feature with distractor, no significant difference was found between the D_C + S + and the D_C + S– condition (*t*(33) = 0.85, *p* = 1.00, Cohen’s *d* = 0.15). Significant differences were found between the D_C + S + and the D_C–S + condition (*t*(33) = –4.45, *p* < 0.001, Cohen’s *d* = –0.76), and between the D_C + S– and the D_C–S + condition (*t*(33) = −6.38, *p* < 0.001, Cohen’s *d* = −1.09).

We further examined whether the attentional disengagement was delayed or accelerated by one-sample *t*-tests comparing the SRT difference scores to zero. The results revealed that significant delay in attentional disengagement in the T_C + S + (*t*(33) = 6.17, *p* < 0.001, Cohen’s *d* = 1.06) and the T_C + S– condition (*t*(33) = 4.02, *p* < 0.001, Cohen’s *d* = 0.69). The attentional disengagement was significantly accelerated in the D_C + S + (*t*(33) = −5.11, *p* < 0.001, Cohen’s *d* = −0.88) and the D_C + S– condition (*t*(33) = −7.27, *p* < 0.001, Cohen’s *d* = −1.25). No significant difference was found in the T_C–S + (*t*(33) = 0.98, *p* = 0.33, Cohen’s *d* = 0.17) and the D_C–S + condition (*t*(33) = −0.91, *p* = 0.37, Cohen’s *d* = −0.16).

After matching the search difficulty between color and shape feature dimensions, we found that there was a significant delay in attentional disengagement when the center stimulus was the same color as the target, regardless of shape. When the center stimulus shared color features with the salient distractor, attention disengagement was accelerated. Moreover, no significant effect on attentional disengagement was found when the center stimulus shared shape features with either the target or the salient distractor. In Experiment 2, we found the differences in the effects of color and shape feature dimensions on attentional disengagement after matching the search difficulty. These results were similar to that in Experiment 1A and suggested that the different effects were caused by the feature asymmetry. Theeuwes (1992) observed that the selectivity of attention in visual search depended on the discernibility of stimulus dimensions. Therefore, in Experiment 3, we improved the salient discernibility of shape dimension in the matching phase, trying to make color and shape equally attractive to participants in the dimensions.

## Experiment 3A

### Method

#### Participants

Thirty-two healthy adults (28 females; age (mean ± SD): 20.44 ± 1.34 years; age range: 18-31 years) participated in Experiment 3A. All participants had normal or corrected-to-normal color vision. Each participant signed an informed consent form.

#### Materials and design

**FIGURE 5:**
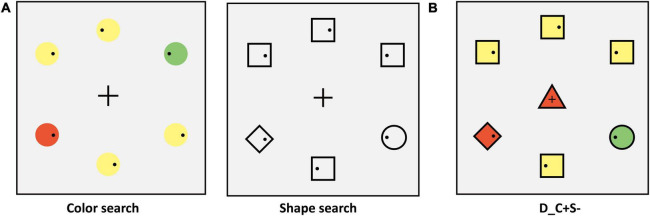
**(A)** Schematic search display of the matching phase (e.g., target: green circle). The schematic procedure of the matching phase is shown in Figure 3. **(B)** Schematic one of the center stimuli conditions, the other conditions were the same as in Experiment 1A. The schematic procedure of the search task phase was similar to that in Experiment 1A, except that the color of stimuli.

In the matching phase, we improved the salient discernibility of shape dimension by adding a black outline to the outer edge of the shape. The difference with Experiment 2 was that we used the black outline as stimuli without color filling in the shape search task. In the matching phase, the average SRT of shape search tasks was calculated for each participant (mean ± SD: 252.69 ± 21.87 ms). We calculated the SRTs of the color search task under different color levels and gained the curve according to Sigmoid curve fitting (y = base + max/(1 + exp((xhalf-x)/rate))). In this formula, the color level was served as x, and SRTs were served as *y*. After Sigmoid curve fitting, we used the curve and served average SRT of the shape search task as y to calculate the color level for each participant (mean ± SD: 13.61 ± 5.05). This color level was used for stimuli in the search task phase and we added the same black outline as the matching phase (examples are shown in Figure 5B).

#### Apparatus and procedure

The apparatus parameters were the same as those in Experiment 1. The experimental procedure was the same as that of Experiment 2.

### Results and discussion

#### Data analysis

About 6.33 ± 7.48% trials were excluded from the analysis because the participant blinked or made a saccade at the time when the search display appeared, or the saccadic latency was less than 80 ms. About 17.40 ± 9.00% trials were excluded from the analysis because the location of the first saccade did not within 2° of the target location. The accuracy of the keypress response was 98.54 ± 1.59%, and the mean RT of correct trials was 0.63 ± 0.05 s. The SRT (ms), reaction time (s), accuracy, and saccadic accuracy for all conditions of participants are shown in Table 4.

**TABLE 4:** Mean saccadic reaction time (ms) and reaction time (s) and accuracy and saccadic accuracy for all condition of Exp. 3A (standard deviations are within parentheses).

	T_C + S +	T_C + S–	T_C–S +	Baseline	D_C + S +	D_C + S–	D_C–S +
SRT (ms)	247 (28)	239 (21)	234 (18)	231 (17)	227 (18)	227 (17)	230 (17)
RT (s)	0.65 (0.05)	0.64 (0.05)	0.63 (0.05)	0.63 (0.05)	0.62 (0.05)	0.62 (0.05)	0.62 (0.05)
Accuracy	0.98 (0.03)	0.99 (0.02)	0.99 (0.02)	0.98 (0.02)	0.99 (0.02)	0.99 (0.03)	0.99 (0.03)
Saccadic accuracy	0.72 (0.16)	0.76 (0.15)	0.76 (0.14)	0.77 (0.15)	0.79 (0.13)	0.77 (0.15)	0.77 (0.13)

#### Saccadic disengagement

We subtracted the SRTs of the baseline condition from the SRT of every other condition. The SRT difference scores were shown in Figure 2E. The 2 (center stimulus type: shared features with the target or salient distractor) × 3 (shared features: both color and shape, color, shape) repeated measures ANOVA (Bonferroni-corrected comparisons) was used to analyze the SRT difference scores. The results revealed main effect of the center stimulus type (*F*(1, 31) = 65.87, *p* < 0.001, η*p*^2^ = 0.68), shared features (*F*(2, 62) = 8.09, *p* < 0.001, η*p*^2^ = 0.21), and their interaction (*F*(1.62, 50.22) = 26.96, *p* < 0.001, η*p*^2^ = 0.47). Further simple-effects analysis (Bonferroni-corrected comparisons) revealed that the SRT difference scores of the T_C + S + condition were significantly longer than that of the T_C + S– and the T_C–S + condition (T_C + S–: *t*(31) = 4.04, *p* < 0.001, Cohen’s *d* = 0.71, T_C–S + : *t*(31) = 5.02, *p* < 0.001, Cohen’s *d* = 0.89). The SRT difference scores of the T_C + S– condition were significantly longer than that of the T_C–S + condition (*t*(31) = 2.70, *p* < 0.05, Cohen’s *d* = 0.48). Significant differences were found between the T_C + S + and the D_C + S + condition (*t*(31) = 7.36, *p* < 0.001, Cohen’s *d* = 1.30), between the T_C + S– and the D_C + S– condition (*t*(31) = 6.54, *p* < 0.001, Cohen’s *d* = 1.16), and between the T_C–S + and the D_C–S + condition (*t*(31) = 3.64, *p* < 0.001, Cohen’s *d* = 0.64). When the center stimulus shared a feature with a salient distractor, the result was the same as that of Experiment 2. No significant difference was found between the D_C + S + and the D_C + S– condition (*t*(31) = –0.38, *p* = 1.00, Cohen’s *d* = –0.07). Significant differences were found between the D_C + S + and the D_C–S + condition (*t*(31) = –5.31, *p* < 0.001, Cohen’s *d* = -0.94), and between the D_C + S– and the D_C–S + condition (*t*(31) = –3.00, *p* < 0.05, Cohen’s *d* = –0.53).

We further examine whether the attentional disengagement was delayed or accelerated by one-sample *t*-tests comparing the SRT difference scores to zero. The results revealed significant delay in attentional disengagement in the T_C + S + (*t*(31) = 5.47, *p* < 0.001, Cohen’s *d* = 0.97), the T_C + S– (*t*(31) = 4.21, *p* < 0.001, Cohen’s *d* = 0.74) and the T_C–S + condition (*t*(31) = 3.48, *p* < 0.01, Cohen’s *d* = 0.61). The attentional disengagement was significantly accelerated in the D_C + S + (*t*(31) = −4.44, *p* < 0.001, Cohen’s *d* = −0.79) and the D_C + S– condition (*t*(31) = −3.70, *p* < 0.001, Cohen’s *d* = −0.65). No significant difference was found in the D_C–S + condition (*t*(31) = −0.43, *p* = 0.67, Cohen’s *d* = −0.08). These results showed that attentional disengagement was delayed when the center stimulus shared features with the target. And the speed of attentional disengagement would accelerate when the center stimulus was the same color as the salient distractor, regardless of shape. The different results between Experiments 3A and 2 were that the influence of shape features on attentional disengagement was found in Experiment 3. This effect occurred only when the center stimulus shared shape features with the target, but it was still smaller than that in color dimension. And these results suggested that although there was a delay in attentional disengagement in the shape dimension, the feature asymmetry still existed.

## Experiment 3B

### Method

#### Participants

Thirty-four healthy adults (28 female; age (mean ± SD): 20.85 ± 2.45 years; age range: 18-27 years) participated in Experiment 3B. All participants had normal or corrected-to-normal color vision. Each participant signed an informed consent form.

#### Materials and design

**FIGURE 6:**
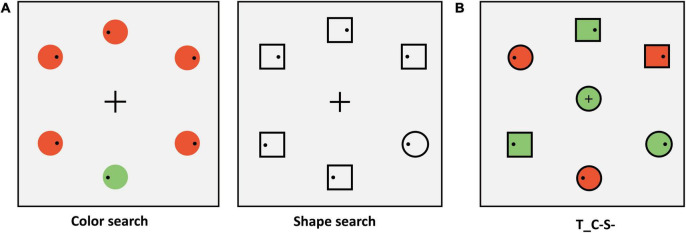
**(A)** Schematic search display of the matching phase (e.g., target: green circle). The schematic procedure of the matching phase was shown in Figure 3. **(B)** Schematic one of the center stimuli conditions, the other conditions, and the Schematic procedure of the search task phase were similar to that in Experiment 1B.

To ensure that the search difficulty of color and shape feature dimensions was identical in the search task phase, we carried out a matching phase before the search task phase. We adopted the single feature search task in the matching phase (see Figure 6A). The participants were required to complete two tasks: A color search task and a shape search task. In the color search task, the peripheral stimuli consisted of six circles (red or green), only one of which had a different color from the others (a red circle among green circles, or vice versa). The target was red or green and varied between subjects. The stimuli colors were red (RGB:175 + x, 175, 175) and green (RGB:175, 175 + x, 175). The color had six levels in the matching phase that correspond to x values of 5, 10, 15, 20, 25, and 30. With the increase of the value of “*x*”, discriminability between different colors increases. In the color search task of the matching phase, the color of each stimulus was randomly selected from the six levels with equal proportions. In the shape search task, the peripheral stimuli consisted of one circle (target) and five squares (distractors) that were presented as black outline without color filling. In the color and shape search task, participants were instructed to ensure the first saccade to the target location as quickly as possible and respond to whether the small black dot (Diameter: 0.26°; RGB: 0, 0, 0) within target was on the left or right side by pressing the left and right arrow keys. We matched the level of color according to the difficulty of the shape. The average SRT of shape search tasks was calculated for each participant (mean ± SD: 249.47 ± 27.46 ms). We adopted the same method and formula as in Experiment 3A to obtain the color level of each participant (mean ± SD: 10.09 ± 4.05). This color level was used for stimuli in the search task phase and we added the same black outline as the matching phase (examples are shown in Figure 6B).

#### Apparatus and procedure

The apparatus parameters were the same as those in Experiment 1. Each participant was required to complete the matching phase before the search task phase. To balance the order effect, each participant took the order of ABBA or BAAB for different tasks. The sequence of displays in a typical trial of the matching phase was shown in Figure 4. In the matching phase, participants completed one practice block of 72 trials in color search and shape search tasks, respectively. The color search task consisted of ten blocks (36 trials in each block) for a total of 360 trials. The shape search task consisted of four blocks (36 trials in each block) for a total of 144 trials. In the search task phase, the number of blocks, total trials, and the search task of participants were identical to those in Experiment 1B.

### Result and discussion

About 5.04 ± 4.20% of trials were excluded from the analysis because the participant blinked or made a saccade at the time when the search display appeared, or the saccadic latency was less than 80 ms. About 43.12 ± 10.88% trials were excluded from the analysis because the location of the first saccade did not within 2° of the target location. The accuracy of the keypress response was 98.67 ± 1.19%, and the mean RT of correct trials was 0.69 ± 0.11 s. The SRT (ms), reaction time (s), accuracy, and saccadic accuracy for all conditions of participants are shown in Table 5.

**TABLE 5:** Mean saccadic reaction time (ms) and reaction time (s) and accuracy and saccadic accuracy for all condition of Exp. 3B (standard deviations are within parentheses).

	T_C + S +	T_C + S–	T_C–S +	Baseline
SRT (ms)	277 (41)	269 (39)	263 (39)	259 (36)
RT (s)	0.70 (0.12)	0.68 (0.11)	0.68 (0.12)	0.68 (0.11)
Accuracy	0.98 (0.02)	0.99 (0.02)	0.98 (0.02)	0.99 (0.02)
Saccadic accuracy	0.54 (0.12)	0.52 (0.12)	0.52 (0.11)	0.50 (0.12)

#### Saccadic disengagement

We subtracted the SRTs of the baseline condition from the SRTs of each condition. These SRT difference scores were shown in Figure 2F. One-way ANOVA of the SRT differences scores revealed significant main effect of the center stimulus type (*F*(1.70, 56.09) = 21.28, *p* < 0.001, η*p*^2^ = 0.39). *Post hoc* comparisons with Bonferroni correction revealed that the SRT differences scores of the T_C + S + condition were significantly longer than the T_C + S– and the T_C–S + condition (T_C + S–: *t*(33) = 3.55, *p* < 0.01, Cohen’s *d* = 0.61, T_C–S + : *t*(33) = 5.76, *p* < 0.001, Cohen’s *d* = 0.99). Significant difference was found between the T_C + S– and the T_C–S + condition (*t*(33) = 3.51, *p* < 0.01, Cohen’s *d* = 0.60). We further examined whether the attentional disengagement was delayed or accelerated by one-sample *t*-tests comparing the SRT difference scores to zero. The results revealed significant delay in attentional disengagement in the T_C + S + condition (*t*(33) = 7.32, *p* < 0.001, Cohen’s *d* = 1.25), the T_C + S– condition (*t*(33) = 7.15, *p* < 0.001, Cohen’s *d* = 1.23) and the T_C–S + condition (*t*(33) = 2.59, *p* < 0.05, Cohen’s *d* = 0.44).

In Experiment 3B, we improved the salient discernibility of shape dimension and matched the color search to it. The results showed that there was a significant delay in attentional disengagement when the center stimulus shared features with the target, regardless of shape or color. Furthermore, the delay in attentional disengagement was greatest when the center stimulus was identical to the target.

## General discussion

In the current study, we adopted the oculomotor disengagement paradigm by manipulating the center stimulus and used saccade latency as an indicator to investigate the effects of different feature dimensions on attentional disengagement. In both single and conjunction feature search tasks (1A and 1B), we found that attentional disengagement was significantly delayed when the center stimulus was the same color as the target, regardless of shape. When the center stimulus shared color features with the salient distractor, attentional disengagement was significantly accelerated. Furthermore, the delay attentional disengagement was greatest when the center stimulus was identical to the target. The result was consistent with the previous research that attentional disengagement was delayed when the fixated stimulus shared color features with the target (Boot and Brockmole, 2010; Blakely et al., 2012; Wright et al., 2015a,b). However, when the center stimulus shared shape features with the target or salient distractor, there was no significant effect on attentional disengagement (Experiment 1). Previous studies indicated that color features would be more easily perceived than other features (e.g., orientation, shape) (Theeuwes, 1991; Moutoussis and Zeki, 1997; Zhuang and Papathomas, 2011).

Therefore, in the following experiments, we matched the search difficulty of color and shape feature dimensions before exploring the attentional disengagement effect. However, no significant attentional disengagement effect was found in shape feature dimensions (Experiment 2). These results suggested that the different effects of color and shape feature dimensions were caused by feature asymmetry. Several studies showed that when the color feature was used in conjunction with other features, the visual system can use it more efficiently than other features (Williams, 1966; Luria and Strauss, 1975; Williams and Reingold, 2001; Hannus et al., 2006). When provided with information about two or three target features, participants generally fixated on stimuli based on color features (Williams, 1966). Some features of visual stimuli had different influences on attention deployment. Plenty of evidence showed that features like color, orientation, and motion could guide search, but the shape was a controversial guiding attribute, some aspects of which can guide attention were not clear (Wolfe and Horowitz, 2004). Theeuwes (1992) observed that the selectivity of attention in visual search depended on the discernibility of stimulus dimensions. Therefore, in Experiment 3 (single and conjunction feature search), we improved the salient of shape dimension and matched the color search to it. In Experiment 3, we found that the attentional disengagement was delayed when the center stimulus shared shape feature with the target, but it was still smaller than that in the color dimension. The results showed that although there was a delay in attentional disengagement in the shape dimension, the feature asymmetry still existed. Blakely et al. (2012) also found the delay attentional disengagement effect in the shape search tasks. The extent to which participants used shape information increased with discriminability (Williams and Reingold, 2001). Therefore, we improved the salient of shape dimension to make it more discriminable. Participants began to utilize shape information, so attentional disengagement was delayed when the center stimulus shared the shape feature with the target in Experiment 3.

The results suggested that the features of the center fixation stimulus could delay or accelerate disengagement of attention. Previous literature had many discussions on the causes of attentional delay disengagement. Some research suggested that attentional delay was because the simultaneous presentation of irrelevant stimuli influences the subsequent deployment of attention (Kahneman et al., 1983; Folk and Remington, 1998). However, Wright et al. (2015a) further explored the function of delayed disengagement, which encourages attention to process similar target stimuli at a deeper level. Boot and Brockmole (2010) proposed three explanations for the causes of delayed attentional disengagement. For the first explanation, they suggested that goal-oriented attention sets were applied to gaze decisions. When the center stimulus matched the current attention set, attention was difficult to disengage from the stimuli or refocus covert attention at fixation. The second was that the presence of target-defining features at fixation might reduce the salience of the peripheral target. According to Wolfe’s Guided Search 2.0 model (Wolfe, 1994), in which competition for representation within these feature maps by multiple stimuli sharing the same feature results in less influence each of these stimuli had on the master activation or salience map (Blakely et al., 2012). The findings of Blakely et al. (2012) did not support this view. Their result showed the feature participants used to locate the target (red) was separate from the information that held attention at the center (the letter). The nature of the center letter should not influence the salience of the red circle, but delayed attentional disengagement was still found when the center letter was similar to the target letter. Another explanation was that the participants might group the target and center stimulus according to the color perception grouping, which made delayed attentional disengagement. There was clear evidence for grouping by common color, but the effect of grouping on the basis of common shape was not obvious (Quinlan and Wilton, 1998). However, Blakely et al. (2012) and our study found the delay attentional disengagement in the shape feature dimension, so this explanation also seems implausible.

We found that the similarity between the center stimulus and the target might influence the speed of attentional disengagement. Specifically, when the center stimulus was more similar to the target, the disengagement of attention from the center stimulus location was slower (after matching difficulty). Leblanc et al. (2008) investigated the effect of target-distractor similarity on the possibility of attention capture and processing on distractor. They found that target-colored distractors elicited a significant N2pc component, while non-target-colored distractors did not generate N2pc component. However, our results showed that the effect of similarity on attentional disengagement seemed to be influenced by the difficulty of different feature dimensions. Therefore, our results were more likely to support that goal-oriented attention sets were the main cause of delayed attentional disengagement. Blakely et al. (2012) provided evidence that top-down goals do indeed modulate the speed with which attention can disengage from an item within the focus of attention. Anderson (2014) showed that a goal-defined target template might be used to guide selection at a crude perceptual level, failing to distinguish between targets and non-targets with similar characteristics. This also explained the delayed attentional disengagement in our study. We speculated that participants not only had target attention sets but also rejected attention sets. The attentional disengagement was delayed when the center stimulus shared features with the target. When the fixed stimulus shared features with the salient distractor, the attentional disengagement was accelerated. We supposed that the participants might have formed a rejection template or rejection attention set about the salient distractor because the peripheral salient distractor was always constant. When the center stimulus shared features with the salient distractor, the rejection template or rejection attentional set could modulate its likelihood of capturing attention and the duration of attention.

Previous studies provided evidence for the delayed disengagement effect (Boot and Brockmole, 2010; Blakely et al., 2012; Wright et al., 2015a,b). However, in our daily lives, we expected the search to be as efficient as possible. Thus, can the speed of disengagement be affected by learning or being trained? The problem is worth studying. Gaspelin et al. (2019) found that proactive inhibition of salient stimulus resulted from multiple trials of experience with the singleton and target. In addition to goal-driven and stimulus-driven selection, some researchers suggested that the characteristics of stimuli noted or ignored in previous trials were also the factors that influenced attentional capture (i.e., selection history) (Maljkovic and Nakayama, 2000; Mechanisms, 2009; Theeuwes, 2019). Vatterott et al. (2017) found that the heterogeneous environment was more conducive to the subjects learning to reject the additional singleton compared with the homogeneous environment, which encourages generalizable learned distractor rejection and proves that experience plays a substantial role in attentional control. A question for future research is whether delayed disengagement effects can be attenuated by learning or being trained, and which environment is more conducive to learning.

In conclusion, we found the attentional disengagement effect in both single and conjunction feature search tasks. The attentional disengagement was delayed when the task-irrelevant stimulus shared features with the target. And the speed of attentional disengagement would accelerate when the center stimulus was the same color as the salient distractor, regardless of shape. The effects of different feature dimensions on attentional disengagement were influenced by feature asymmetry.

## Data availability statement

The raw data supporting the conclusions of this article will be made available by the authors, without undue reservation.

## Ethics statement

The studies involving human participants were reviewed and approved by Ethics Committee of School of Educational Science, Minnan Normal University, China. The patients/participants provided their written informed consent to participate in this study.

## Author contributions

YH and QZ conceived, designed the experiment, performed the experiments, and wrote the manuscript. YH, QZ, and MS analyzed the data. ZW revised the manuscript critically for important intellectual content. QZ provided technical guidance and site support. All authors contributed to the article and approved the submitted version.
